# Cell-Penetrating Chaperone Nuc1 for Small- and Large-Molecule Delivery Into Retinal Cells and Tissues

**DOI:** 10.1167/iovs.65.8.31

**Published:** 2024-07-19

**Authors:** Binit Kumar, Manish Mishra, Deepa Talreja, Siobhan Cashman, Rajendra Kumar-Singh

**Affiliations:** 1 Department of Developmental, Molecular and Chemical Biology, Tufts University School of Medicine, Boston, Massachusetts, United States

**Keywords:** protein delivery, cell-penetrating peptide, macropinocytosis

## Abstract

**Purpose:**

There are currently no means available for the efficient delivery of recombinant proteins *into* retinal cells in vivo. Although cell-penetrating peptides have been somewhat effective in protein delivery to the retina, they generally require conjugation chemistry with the payload, negatively impacting function of the therapeutic protein. In this study, we developed a novel peptide (Nuc1) that acts as a chaperone for delivery of small and large molecules, including steroids, peptides, antibodies, recombinant proteins, and viruses (adeno-associated viruses [AAVs]) across biological membranes in vivo without the need for conjugation.

**Methods:**

Nuc1 peptide was designed based on sequences known to bind heparan sulfate proteoglycans and nucleolin found on the surface of retinal cells. Nuc1 was injected into the vitreous of mice with a variety of molecules and retinas examined for uptake and function of these molecules.

**Results:**

Nuc1 engages the process of macropynocytosis for cell entry. The delivery of functional recombinant X-linked inhibitor of apoptosis protein to photoreceptors via the intravitreal route of injection inhibited retinal apoptosis. Nuc1 was found to enhance the delivery of anti-VEGF antibodies delivered intravitreally or topically in models of age-related macular degeneration (AMD). Nuc1 enhanced delivery of decorin, facilitating significant inhibition of neovascularization and fibrosis in a model of AMD. Finally, Nuc1 was found to enhance penetration of retinal cells and tissues by AAV via both the subretinal and intravitreal routes of injection.

**Conclusions:**

Nuc1 shows promise as a novel approach for the delivery of recombinant proteins *into* retinal cells in vivo.

The plasma membrane functions as a barrier for the translocation of macromolecules into the cytoplasm. Thus, despite the availability of a library of potentially therapeutic recombinant proteins for the treatment of retinal disease, there is currently no method available for the delivery of such proteins *into* cells in vivo. In order to overcome this barrier, there is significant interest in the development of a class of molecules known as cell-penetrating peptides (CPPs).^1^ A variety of CPPs have been previously shown to be able to be conjugated to heterologous molecules and deliver them across the plasma membrane in vitro and in vivo*.*^2^ CPPs may be designed with consideration of the biological properties of specific cells or tissues. For example, we have previously described a CPP termed POD (peptide for ocular delivery) that was modeled upon the glycosaminoglycan-binding regions of proteins abundantly present in the retina, specifically, acidic and basic fibroblast growth factor (bFGF).^3^ Competition studies indicated that the cell-penetrating properties of POD were significantly dependent upon the levels of heparan sulfate proteoglycans (HSPGs) on the surface of cells,^3^ highlighting HSPGs on retinal tissues as candidate moieties for CPP-mediated delivery of macromolecules into retinal cells.

Similar to bFGF, VEGF plays a key role in retinal homeostasis.^4^^,^^5^ The VEGFA165 isoform of VEGF contains a highly basic domain that allows this isoform to interact with and localize to the heparan sulfate–rich extracellular matrix.^6^ A VEGFA splice variant, VEGFA165b from kidney epithelial cells is identical to VEGFA165 except for the last six amino acids. VEGFA165b and VEGFA165 bind VEGF receptors 1 and 2 with similar affinity. However, VEGFA165b only weakly binds to heparan sulfate,^7^ implicating the C-terminus of VEGF165 in heparan sulfate binding.

Laminin is a large basement membrane glycoprotein of the retinal extracellular matrix. The laminins influence tissue development, cell differentiation, migration, and adhesion.^8^ Laminin also binds heparan sulfate proteoglycans, and it mediates cellular interactions with basal lamina. A region proximal to the carboxyl globule of laminin 1 is an active site for cell adhesion.^9^ Interestingly, this region also binds to nucleolin,^10^ a protein that is typically found in the nucleus and on the surface of rapidly dividing cells,^11^^–^^13^ but it is also unexpectedly found in the retina, including terminally differentiated cells such as photoreceptors.^14^ We have previously found that nucleolin-binding molecules may be utilized to deliver macromolecules into retinal cells in vivo*.*^15^^–^^18^

Based on these observations, we hypothesized that a novel peptide composed of the nucleolin-binding region from laminin 1, fused with the VEGFA165-binding region of HSPGs via a flexible polyglycine linker, termed Nuc1, may have combined cell-binding and cell-penetrating properties ideal for use in the retina. Such a CPP may be potentially conjugated to small and possibly large molecules to facilitate macromolecular delivery into retinal cells.

HSPGs have been implicated in internalization of macromolecules in a clathrin- and caveolin-independent pathway, instead, involving macropinocytosis.^19^ Macropinocytosis is characterized by actin-driven membrane ruffling and can result in internalization of extracellular fluids during formation of large intracellular vacuoles. A variety of bacteria and viruses can induce macropinocytosis for cell entry.^20^ This natural process lends itself to the bystander delivery of macromolecules across biological membranes. In this study, we present evidence that Nuc1 can deliver a variety of therapeutically relevant molecules into retinal cells and tissues. Importantly, Nuc1 could exhibit these properties without the need for chemical conjugation with its cargo and thus it acted akin to a chaperone instead of a prototypical CPP for transmembrane transport for macromolecules.

## Materials and Methods

### Reagents

5′ 6-FAM (Fluoroscein) labeled or unlabeled Nuc1 peptide with the sequence ASIKVAVSAGGDKPRR or the scrambled sequence RASIAKARDVKGVPGS referred to as SC was synthesized and purified by Biomatik (Wilmington, Delaware, USA). Primary antibodies used in this study are against glutamine synthetase (Müller cells, cat. ab73593; Abcam, Cambridge, MA, USA), anti–β-III-tubulin (ganglion cells, cat. ab18207; EMD Millipore, Burlington, MA, USA), anti-rhodopsin (rod cells, kind gift from Dr. Robert Molday), anti–S-opsin (cone cells, cat. AB5407; EMD Millipore), anti-PKCα (bipolar cells, cat. MA1-157; Invitrogen, Carlsbad, CA, USA), anti-VEGF antibody (cat. AF-493-NA; R&D Systems, Minneapolis, MN, USA), or a–smooth muscle actin (α-SMA) antibody (cat. C6198; Sigma, St. Louis, MO, USA). Fluorescein-labeled dexamethasone (cat. D1383) was purchased from Invitrogen. Recombinant human X-linked inhibitor of apoptosis protein (XIAP, cat. 822XF050) was purchased from R&D Systems. Recombinant decorin (cat. SRP6454) was purchased from Millipore Sigma (Burlington, MA, USA).

### Animals

This study was carried out in accordance with the Statement for the Use of Animals in Ophthalmic and Vision Research, set out by ARVO, and was approved by Tufts University Institutional Animal Care and Use Committee. Six- to 8-week-old C57BL/6J mice were purchased from Jackson Laboratory (Bar Harbor, ME, USA) and maintained under a 12-hour light/dark cycle.

### Intraocular Injections

Mice were anesthetized by intraperitoneal injection of a mixture containing ketamine (100 mg/kg; Phoenix, St. Joseph, MO, USA) and xylazine (10 mg/kg; Lloyed, Shenandoah, IA, USA), followed by topical application of 0.5% proparacaine hydrochloride (Akorn Inc., Lake Forest, IL, USA) to the cornea. Intravitreal or subretinal injections were performed using a 33-gauge needle and a 5- µL glass syringe. Eyes were enucleated and fixed in 4% paraformaldehyde (PFA) for 4 to 6 hours. Retinal cryosections (14 µm) were taken using a Microm HM 550 cryostat (Kalamazoo, MI, USA).

### Immunohistochemistry

Retinal cryosections (14 µm) were rehydrated in phosphate-buffered saline (PBS) for 15 minutes, permeabilized, and blocked with 6% normal goat serum in PBS containing 0.1% Triton-X-100 (or using mouse-on-mouse kit as appropriate) for 45 minutes and incubated overnight in a saturated chamber with the primary antibodies against anti-glutamine synthetase (Müller cells), anti–β-III-tubulin (ganglion cells), anti-rhodopsin (rod cells), anti–S-opsin (cone cells), and anti-PKCα (bipolar cells). Subsequently, sections were washed and incubated with respective secondary antibodies labeled with Alexa Fluor 594 or 488 (Molecular Probes, Eugene, OR, USA) or Cy3 (Jackson Immunoresearch, Westgrove, PA, USA). Slides were mounted in antifade medium containing DAPI (Vectashield-DAPI; Vector Laboratories, Burlingame, CA, USA) to counterstain nuclei, and images were captured with a Leica TCS SPE microscope (Leica Microsystems, Wetzlar, Germany).

### N-methyl-nitrosourea–Induced Retinal Apoptosis

Mice were injected intravitreally with recombinant XIAP or with XIAP in combination with Nuc 1. After 4 hours, mice were injected with 50 mg/kg N-methyl-nitrosourea (MNU) intraperitoneally. After 24 hours, eyes were harvested and cryosectioned as described above. To detect apoptotic cells, the TUNEL method was performed on cryosections using the In Situ Cell Death Detection Kit, TMR Red (Millipore Sigma), as per the manufacturer's instructions. The sections were imaged and used for quantification of TUNEL-positive cells using ImageJ (FIJI version and plug in) as described previously.^21^

### Retinal Detachment

Retinal detachment was induced by subretinal injection of 3 µL (10 mg/mL) sodium hyaluronate (Healon, Advanced Medical Optics, Santa Ana, CA, USA) using a 32-gauge needle (Becton-Dickinson, Franklin Lakes, NJ, USA) and a 5-µL glass syringe (Hamilton, Reno, NV, USA). Twenty-four hours after injection, animals were injected intravitreally with XIAP (1.4 µg) coformulated with Nuc1 (0.4 µg). A total of 72 hours after retinal detachment, eyes were enucleated and cryosections taken using a Microm HM 550 cryostat and processed as above for TUNEL analysis.

### Laser-Induced Choroidal Neovascularization

Four laser spots per eye were created around the optic nerve head using an argon laser set at 330 mW power and a 100-ms pulse. Immediately after the laser burn, mice were injected once intravitreally with 0.3 ng murine anti-VEGF antibody with or without 1 µg Nuc1 peptide. In a separate group, mice received**,** by topical application to the cornea**,** a mixture of 1.8 µg anti-VEGF antibody with or without 4 µg Nuc1 twice daily for 10 days. After 7 and 10 days of intravitreal and topical application**,** respectively, eyes were enucleated, retinas and corneas removed, and eyecups (RPE/choroid) fixed in 4% PFA. The RPE/choroid tissue was incubated with α-SMA antibody at 4°C overnight, followed with Cy3-labeled secondary antibody along with fluorescein–Griffonia Simplicifolia Lectin I (GSL I; 10 mg/mL in PBS; Vector Laboratories) for 2 hours at room temperature. Fluorescein–GSL I–stained choroidal neovascularization (CNV) lesions or Cy3-stained fibrotic spots were imaged on the RPE/choroid using a fluorescence microscope and the area of neovascularization and fibrosis were quantified using ImageJ software (http://rsbweb.nih.gov/ij/).

### Retinal Transduction by AAV2/9

Green fluorescent protein (GFP) mRNA expression in eyes injected intravitreally with AAV9CAGGFP with and without Nuc1 was quantified by real-time PCR (RT-qPCR). Two weeks following injection, total RNA from the retina was isolated using RNeasy Mini Kit (Qiagen, Valencia, CA, USA), and cDNA was synthesized using the one-step high-capacity cDNA reverse transcription kit (Applied Biosystems, Foster City, CA, USA). GFP expression was quantified using a SYBR Green (Applied Biosystems) assay on the BioRad thermocycler system (iQ5 Multicolor real-time PCR detection system).

### Retinal Explant Culture

After careful removal, the eyeballs from C57BL6/J mice were transferred to a Petri dish containing fresh cold Dulbecco's modified Eagle's medium (DMEM). Under a dissecting microscope, the cornea, ocular muscles, and lens were removed and flat mounts prepared via making four incisions from the margin of the eye cup toward the optic nerve. Flat mounts were transferred into six-well plates containing fresh DMEM/F12 mixed with 5′ 6-FAM-Nuc1 or 5′ 6-FAM-SC (1 µg/mL) in presence or absence of 5 µM imipramine (Sigma). After 4 hours of culturing in a cell culture incubator (37°C, 5% CO_2_), retinas were washed twice with PBS and fixed with 4% PFA for 15 minutes. Thereafter, the tissue sections were soaked in 15% to 30% sucrose at 4°C overnight. Retinal explants were frozen into Tissue-Tek cryomolds (Torrence, CA, USA) and the 14-µm sections were imaged under a Leica (Teaneck, NJ, USA) TCS-SPE confocal microscope.

### Statistical Analysis

All data are presented as mean ± SD. All statistical analysis was performed by unpaired Student's *t*-test using Prism 5 (GraphPad Software, La Jolla, CA, USA), except where indicated. Studies measuring TUNEL-positive cells in the retina following MNU injection used measurements from 20 different regions, randomly selected from five retinal sections per eye (*n* = 4), and analyzed using Fiji version of ImageJ and a plug-in as described previously.^21^ One-way ANOVA analysis was used to quantify the effect of intravitreal anti-VEGF in laser-induced CNV.

## Results

### Nuc1 Peptide Penetrates Retinal Tissues and Cells Following Intravitreal Injection

**Figure 1.**
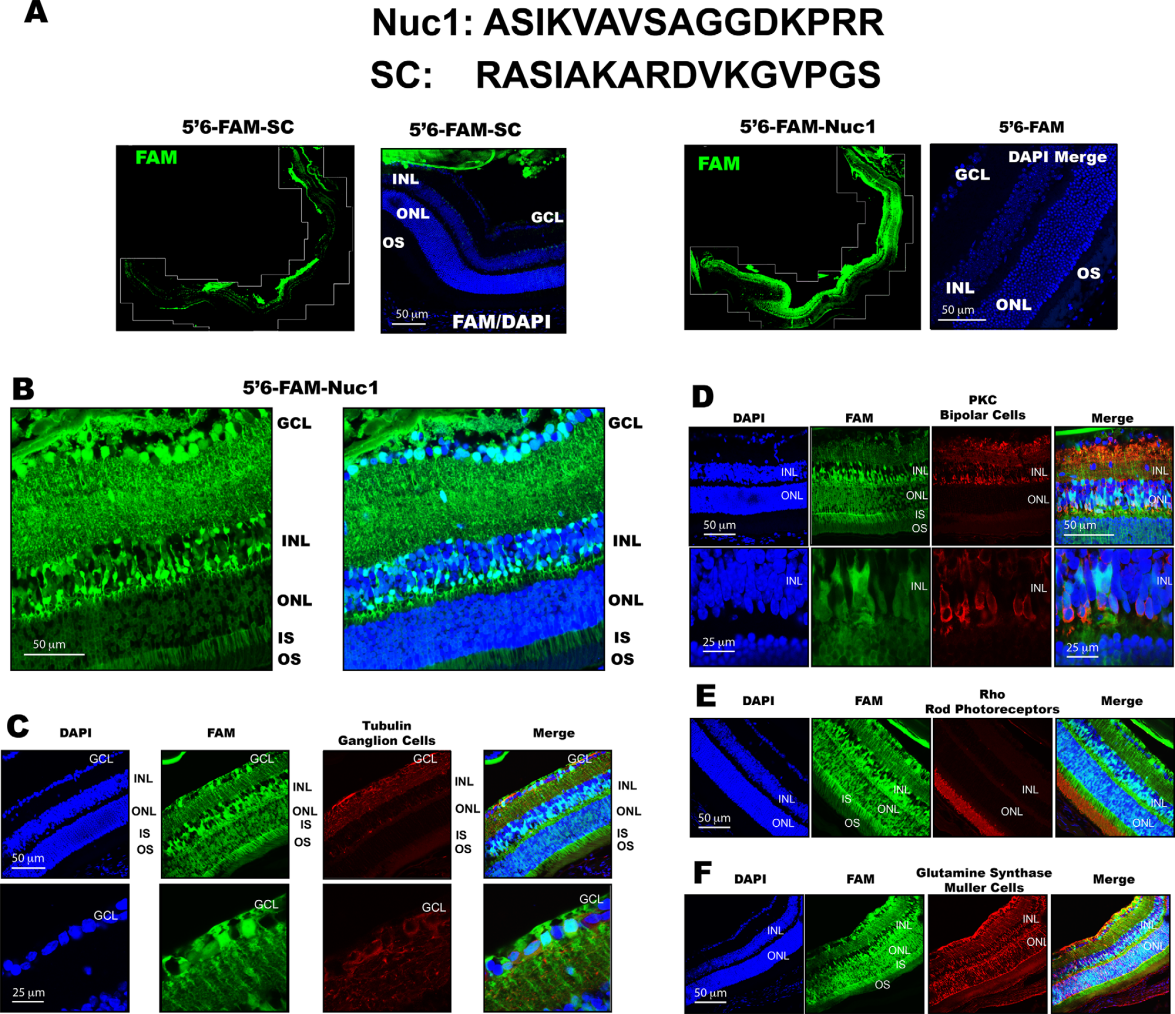
Nuc1 peptide penetrates retinal tissues and cells following intravitreal injection. (**A**) Sequence of Nuc1 and SC (scrambled) peptides with montage of representative retinal cryosection of mouse injected intravitreally with 5′ 6-FAM-Nuc1 or 5′ 6-FAM-SC, indicating that whereas 5′ 6-FAM-SC does not significantly penetrate the retina, 5′ 6-FAM-Nuc1 localizes to all layers of the retina, including GCL, INL, IS, and OS following intravitreal injection. DAPI/5′ 6-FAM (FAM) overlay is also shown. (**B**) Higher-magnification image of 5′ 6-FAM-Nuc1 injected eyes. (**C–E**) Representative retinal cryosections of mice injected intravitreally with 5′ 6-FAM–labeled Nuc1 immunostained with antibodies against tubulin (**C**), PKC (**D**), rod opsin (**E**), and glutamine synthase (**F**). For some panels**,** higher-magnification images are also presented for detail. Each section is a representative example from *n* = 4 to 5 animals per group. DAPI, 4′,6-diamidino-2-phenylindole; IS, inner segments; OS, outer segments.

In order to determine whether Nuc1 peptide was toxic to the murine retina, we injected various amounts (0.5–4.0 µg) 5′ 6-FAM–labeled Nuc1 into the vitreous of 6-week-old C57BL/6J mice and processed the retinas as described below. We determined that 1  µg Nuc1 was not toxic to the retina, whereas 4.0 µg Nuc1 generated some toxicity (data not shown). Based on these observations, we selected 1  µg as an optimal working dose. We then injected 1  µg 5′ 6-FAM–labeled Nuc1 suspended in 1  µL H_2_O or 1  µg 5′ 6-FAM-SC (a scrambled peptide) into the vitreous of 6-week-old C57BL/6J mice. Four hours postinjection, eyes were enucleated, fixed, and cryosectioned and the retina was imaged by fluorescence microscopy. We found that whereas 5′ 6-FAM-SC accumulated primarily at the inner limiting membrane and was very weakly visible in the retina (Fig. 1A), FAM-labeled Nuc1 was abundantly localized to all layers of the retina, including the ganglion cell layer (GCL), inner nuclear layer (INL), outer nuclear layer (ONL), inner segments, and outer segments (Figs. 1A, 1B). Costaining of 5′ 6-FAM-Nuc1 cryosections with antibodies targeting tubulin (Fig. 1C), protein kinase C (PKC) (Fig. 1D), rod opsin (Fig. 1E), or glutamine synthase (Fig. 1F) revealed that Nuc1 targeted ganglion cells, bipolar cells, photoreceptors, and Müller cells, respectively. We conclude that Nuc1 peptide penetrates the retina and labels a variety of retinal cells following intravitreal injection.

### Nuc1 Facilitates Recombinant Protein Penetration Into Retinal Cells

**Figure 2.**
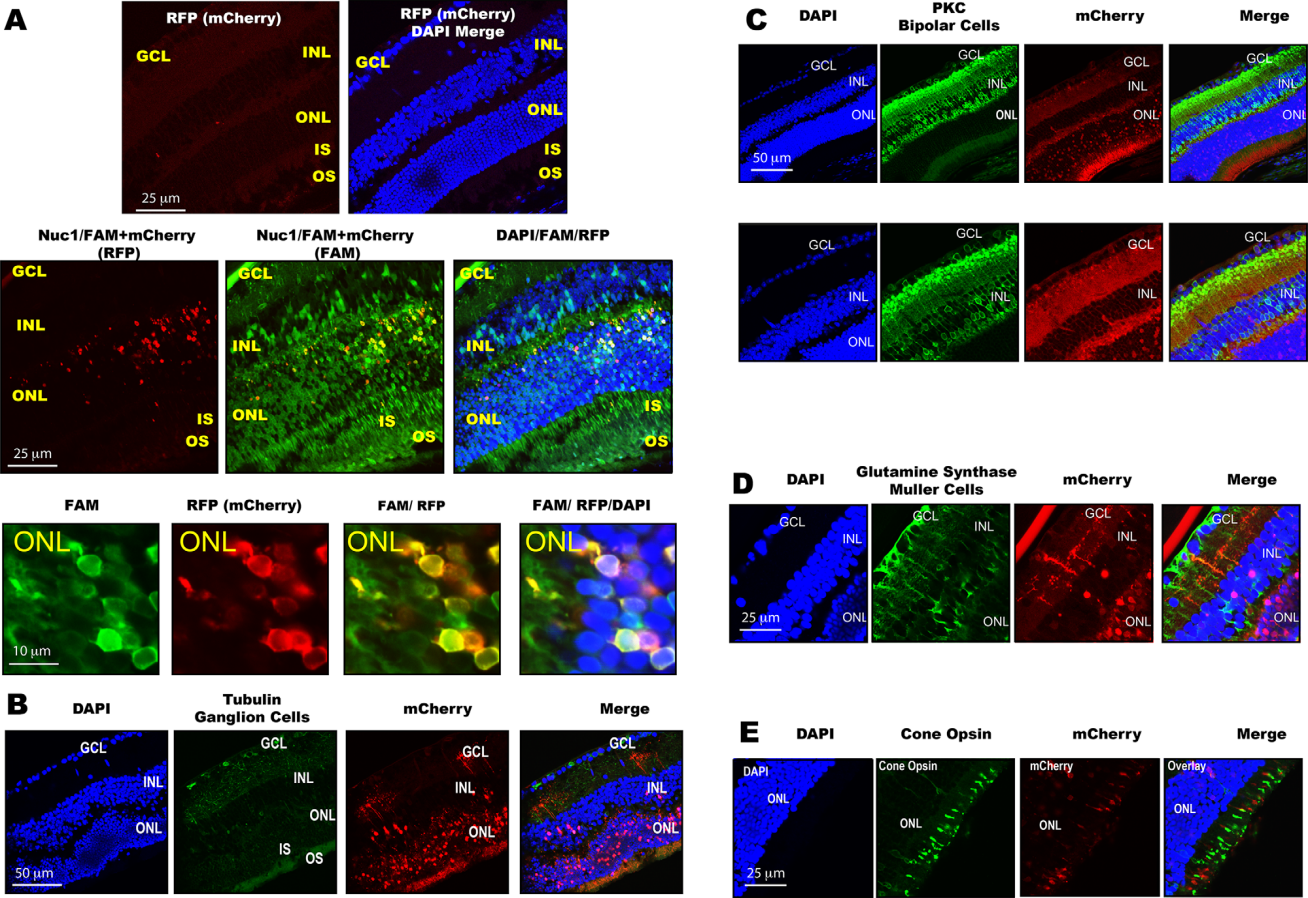
Nuc1 facilitates protein delivery into retinal cells. (**A**) Representative retinal cryosection of mouse injected intravitreally with mCherry only or coinjected with mCherry and 5′ 6-FAM–labeled Nuc1 (Nuc1/FAM) shows significant uptake of mCherry into a variety of retinal cell types, predominantly in the ONL. Higher magnification of the ONL is also presented. There is no significant uptake of mCherry into the retina without the use of Nuc1. (**B–E**) Retinal cryosections from mice coinjected intravitreally with mCherry and nonlabeled Nuc1 and stained using antibodies against tubulin to identify ganglion cells (**B**), PKC for bipolar cells (**C**), glutamine synthase for Müller cells (**D**), or cone opsin for photoreceptors (**E**). Each section is a representative example from *n* = 4 to 5 animals per group.

We hypothesized that Nuc1 may be utilized to deliver heterologous proteins into retinal cells and tissues. We also hypothesized that through the process of macropinocytosis, heterologous proteins may be coinjected with Nuc1 instead of having to be chemically linked. In order to test these hypotheses, we coinjected 4  µg of a recombinant red fluorescent protein (mCherry) with 1  µg 5′ 6-FAM–labeled Nuc1 into the vitreous of 6-week-old C57BL/6J mice. As a negative control, we injected 4  µg mCherry alone. After 4 hours, tissues were processed as described above. We found that whereas there was no significant penetration of mCherry into retinal tissues or cells, mCherry coinjected with 5′ 6-FAM–labeled Nuc1 exhibited significant uptake of mCherry into a variety of retinal cells (Fig. 2A) and most abundantly in the ONL.

When the 5′ 6-FAM–labeled Nuc1 was substituted with unlabeled Nuc1 and coinjected with mCherry, robust uptake of mCherry was still observed, demonstrating that the red fluorescence signal was not bleed-through from the FAM channel (Figs. 2B–E). Costaining of retinal sections with antibodies targeting tubulin (Fig. 2B), PKC (Fig. 2C), glutamine synthase (Fig. 2D), or cone opsin (Fig. 2E) revealed that mCherry was localized to ganglion cells, bipolar cells, Müller cells, and cone photoreceptors, respectively. Relative to other cell types, ganglion cells were only found to be minimally receptive for uptake of mCherry. Furthermore, perhaps due to the small size of the mouse eye or the inherent properties of Nuc1, there was some variability observed between eyes. However, all retinas exhibited the strongest mCherry signal in the ONL.

### Nuc1 Facilitates the Delivery of Functional Antiapoptotic Proteins Into the Retina

Next, we sought to investigate whether Nuc1 could facilitate *internalization* of heterologous proteins into retinal cells in vivo. Programmed cell death, or apoptosis, is a common pathway activated as a consequence of retinal degeneration in diseases such as retinitis pigmentosa.^22^ Intraperitoneal injection of MNU in mice causes induction of apoptosis in the ONL, and thus, this model is routinely utilized for testing the efficacy of inhibitors of apoptosis.^23^ Adult (6- to 8-week-old) C57BL/6J mice were injected intravitreally either with 1.4  µg recombinant human XIAP alone or with 1.4  µg XIAP coinjected with 0.4 µg Nuc1. After 4 hours, mice were injected intraperitoneally with 50 mg/kg MNU. To determine the background level of apoptosis in the retina, a group of mice was injected intraperitoneally with PBS. After an additional 24 hours, eyes of mice were enucleated and processed as above. To detect apoptotic cells, the TUNEL method was performed on retinal cryosections and TUNEL-positive cells quantified using ImageJ software as described previously.^21^

**Figure 3.**
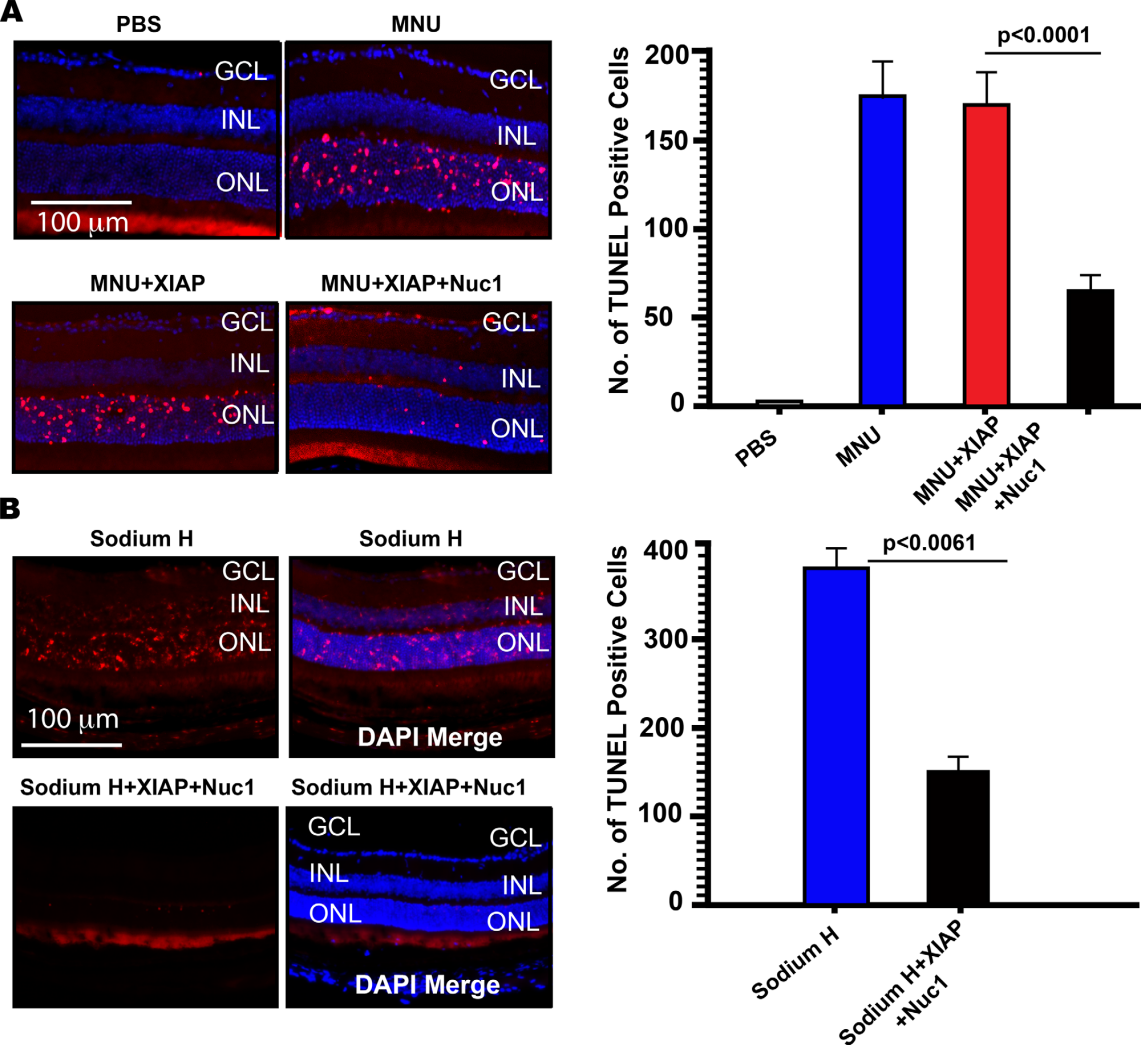
Protein function is retained following intravitreal Nuc1 delivery of protein. (**A**) Representative TUNEL-stained retinal cryosections from mice injected intraperitoneally with MNU. Quantitation of TUNEL-stained retinal cells for each group of mice shows that Nuc1 coinjected with XIAP confers significant (65.7%, *P* < 0.0001) protection against MNU-induced apoptosis (TUNEL-positive cells) relative to untreated MNU-injected mice, while MNU-injected mice intravitreally injected with XIAP alone showed no significant reduction in apoptosis (TUNEL-positive cells; 2.2%, *P* = 0.9902) relative to untreated MNU-injected mice. (**B**) Representative TUNEL-stained retinal cryosections from mice injected in the subretinal space with sodium hyaluronate (Sodium H), a model of retinal detachment. Quantitation of TUNEL-stained retinal cells shows that Nuc1 coinjected with XIAP confers a significant (60%, *P* = 0.0061) reduction in apoptosis (TUNEL-positive cells) relative to mice injected with Sodium H alone. Representative images are randomly selected sections obtained from *n* = 3 to 4 animals per group. Data are presented as mean ± SD and was analyzed using an unpaired *t*-test and the data were considered significant at *P* < 0.05.

As anticipated, intraperitoneal injection of MNU induced extensive apoptosis in the ONL, as evident from the high number of TUNEL-positive cells in this group of animals relative to PBS-injected animals (Fig. 3A). Eyes injected intravitreally with XIAP protein *only* prior to MNU injection exhibited no significant reduction (2.2%, *P* = 0.9902) in the number of TUNEL-positive cells relative to MNU (Fig. 3A), that is, XIAP injected intravitreally by itself did not significantly attenuate apoptosis in the ONL. In contrast, mice that were injected with recombinant human XIAP protein in combination with Nuc1 exhibited a significant reduction (65.7%, *P* < 0.0001) in the number of TUNEL-positive cells relative to XIAP alone. We thus conclude that Nuc1 enables *functional* XIAP protein to penetrate retinal cells and enable inhibition of apoptosis in the ONL.

To further extend these observations, we studied XIAP-mediated reduction of apoptosis associated with retinal detachment. Detachment of the retina results in retinal cell apoptosis within the ONL and INL.^24^ We generated a detachment of the retina by injection of 3 µL (10 mg/mL) sodium hyaluronate into the subretinal space. One day following retinal detachment, animals were coinjected intravitreally with 1.4 µg XIAP and 0.4 µg Nuc1, and 72 hours later, eyes were fixed and stained for TUNEL as described above. We found that there was an approximately 60% (*P* = 0.0061) reduction in the number of TUNEL-positive cells in sodium hyaluronate–injected mice, when XIAP was coinjected with Nuc1, relative to mice injected with sodium hyaluronate alone (Fig. 3B).

### Nuc1 Facilitates the Delivery of Antibodies Into the Retina

In order to expand our observations, we next examined whether Nuc1 could enhance the delivery of antibodies. The most common cause of blindness in the elderly is due to age-related macular degeneration (AMD).^25^ Recombinant proteins or antibodies targeting VEGF injected into the vitreous are US Food and Drug Administration–approved therapies for the treatment of neovascularization associated with the wet form of AMD.^26^ We examined whether Nuc1 could enhance the delivery of VEGF antibody into retinal tissues following intravitreal injection. Laser-induced CNV was generated in adult (6- to 8-week-old) C57BL/6J mice through four laser spots around the optic nerve head as described previously.^27^ Mice were subsequently injected intravitreally with 0.3 ng anti-VEGF antibody only or coinjected with 0.3 ng anti-VEGF antibody in combination with 1 µg Nuc1 or 1 µg Nuc1. The amount of anti-VEGF antibody utilized in these studies was determined through pilot studies (3 µg to 0.3 ng anti-VEGF; data not shown) in order to determine a dose of anti-VEGF antibody that failed to significantly inhibit laser-induced CNV. At 7 days after laser photocoagulation, eyes were harvested and RPE/choroid flat mounts stained with fluorescein–GSL I. CNV lesions were imaged by fluorescence microscopy and the area of the fluorescein-stained lesions quantified.

**Figure 4.**
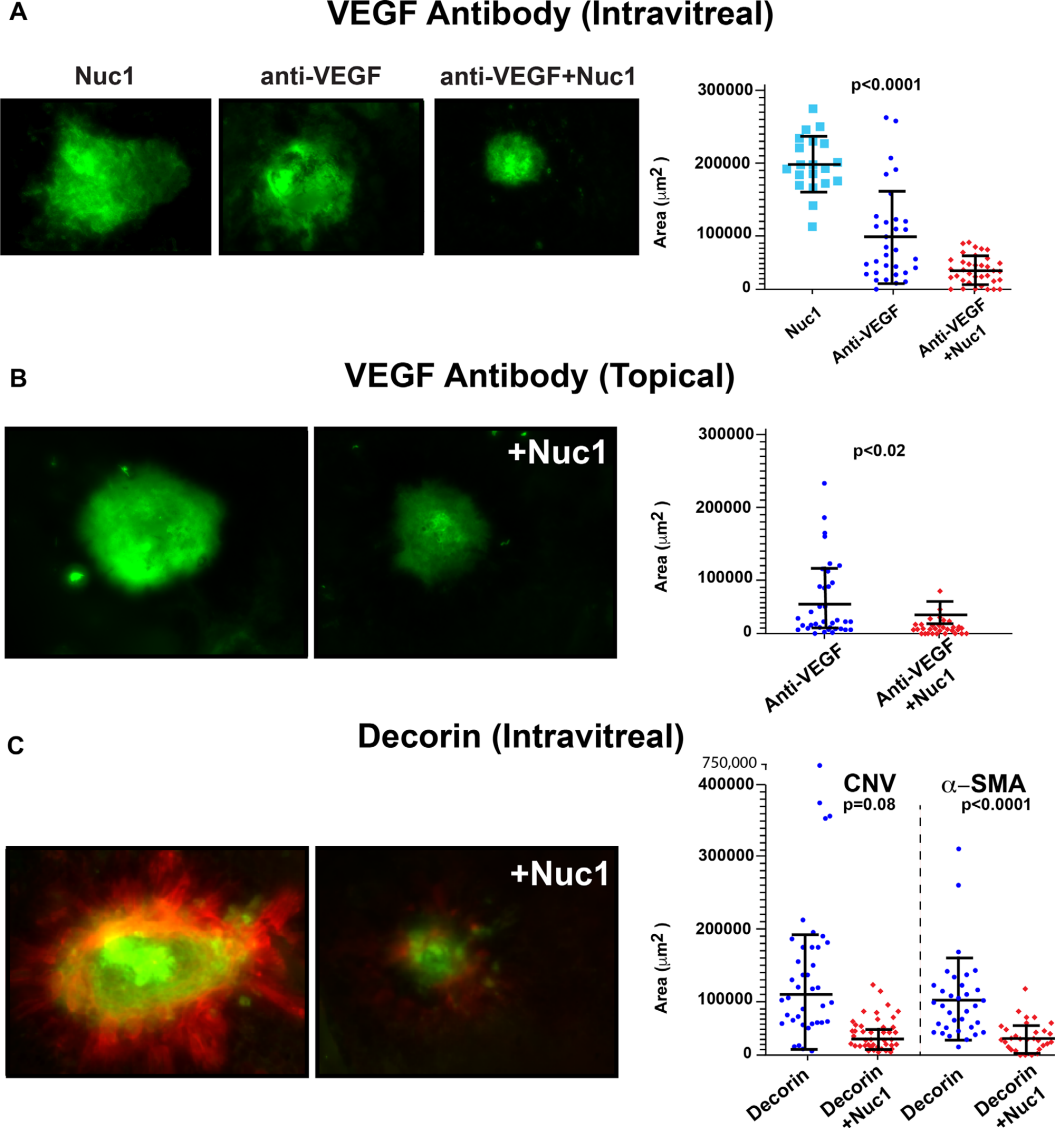
Nuc1 enhances the therapeutic efficacy of antibodies and proteoglycans following intravitreal injection. (**A**) Representative images of fluorescein–GSL I–stained CNV lesions in laser-treated mice at 7 days after intravitreal injection with Nuc1, anti-VEGF antibody alone, or anti-VEGF with Nuc1. Quantitation of the area of CNV lesions reveals that relative to Nuc1, there is a significant reduction (57%, *P* < 0.001) in the size of CNV lesions in eyes injected with anti-VEGF antibody. Furthermore, relative to anti-VEGF antibody alone, there is a significant reduction (>60%; *P* < 0.0001) in the size of CNV lesions in eyes coinjected with anti-VEGF antibody and Nuc1. (**B**) Representative images of fluorescein–GSL I–stained CNV lesions in laser-treated mice following 10 days of twice-daily topical application to the cornea of anti-VEGF antibody alone or anti-VEGF with Nuc1. Quantitation of the area of CNV lesions showed that relative to laser-treated mice treated topically with anti-VEGF antibody alone, topical application of anti-VEGF and Nuc1 further reduced the size of the CNV lesions (∼60%, *P* < 0.02). (**C**) Representative images of fluorescein–GSL I (*green*) and anti–α-SMA (*red*) stained CNV lesions in laser-treated mice at 5 days after intravitreal injection with decorin alone or decorin with Nuc1. Coinjection of Nuc1 with decorin reduced both choroidal neovascularization and fibrosis by 80% (*P* = 0.082) and 73% (*P* < 0.0001)**,** respectively**,** relative to injection of decorin *only*. Data are represented as mean ± SD of the values obtained from 4 to 5 animals/group and significance was analyzed among the groups using an unpaired *t*-test and one-way ANOVA.

Relative to Nuc1 alone, there was a significant reduction (57%, *P* < 0.001) in the size of CNV lesions in eyes injected with anti-VEGF antibody (Fig. 4A). Importantly, relative to anti-VEGF antibody alone, there was a significant reduction (>60%; *P* < 0.0001) in the size of CNV lesions in eyes coinjected with anti-VEGF antibody and Nuc1 (Fig. 4A). We thus conclude that intravitreal coadministration of Nuc1 with anti-VEGF antibody significantly enhances the penetration and efficacy of the anti-VEGF antibody in laser-induced CNV.

We next examined whether Nuc1 could enhance the potency of topically applied anti-VEGF antibody. Following laser-induced CNV, anti-VEGF antibody or anti-VEGF antibody in combination with Nuc1 was applied to the cornea as a suspension twice daily for 10 days. Due to the significantly limited penetration of antibodies into the eye and sclera/choroid when applied topically, in this study, we utilized a significantly higher dose of antibody relative to intravitreal injection, specifically, 1.8 µg anti-VEGF antibody alone or the same amount of anti-VEGF antibody in combination with 4 µg Nuc1. We found that Nuc1 significantly enhanced the efficacy of topically applied anti-VEGF antibody, resulting in ∼60% (*P* < 0.02) reduction in the size of laser-induced CNV (Fig. 4B) relative to anti-VEGF antibody alone. In conclusion, Nuc1 significantly enhances the potency of topically applied antibody in the laser-induced model of wet AMD.

### Nuc1 Enhances the Delivery of Proteoglycans Into the Retina

Antibodies or proteins targeting VEGF have had a very significant clinical impact on the treatment of wet AMD. However, many patients fail to respond to anti-VEGF and, of those individuals who do respond, continue to lose vision, in part due to fibrosis underlying the retina.^28^^,^^29^ It is hypothesized that TGF-b*–*induced epithelial-to-mesenchymal transition of RPE cells underlies pathophysiology of fibrosis associated with AMD.^29^^–^^31^ Decorin is a multifunctional proteoglycan capable of sequestering both VEGF and TGF-β in the extracellular matrix.^32^^–^^34^ We wished to test the hypothesis that Nuc1-mediated delivery of decorin into the retina could inhibit both laser-induced CNV as well as fibrosis. Following laser-induced CNV as described above, 0.5 µg decorin or a similar amount of decorin in combination with 0.5 µg Nuc1 was injected into the vitreous of mice. Five days later, eyecups were harvested and RPE/choroid flat mounts costained with fluorescein–GSL I (for CNV) or an antibody against α–smooth muscle actin (α-SMA) (for fibrosis). We found that coinjection of Nuc1 with decorin reduced both choroidal neovascularization and fibrosis by 80% (*P* = 0.082) and 73% (*P* < 0.0001)**,** respectively**,** relative to injection of decorin only (Fig. 4C). We conclude that Nuc1 can significantly enhance the penetration and efficacy of decorin in a murine model of wet AMD.

### Nuc1 Enhances the Delivery of Peptides and Small Molecules Into the Retina

**Figure 5.**
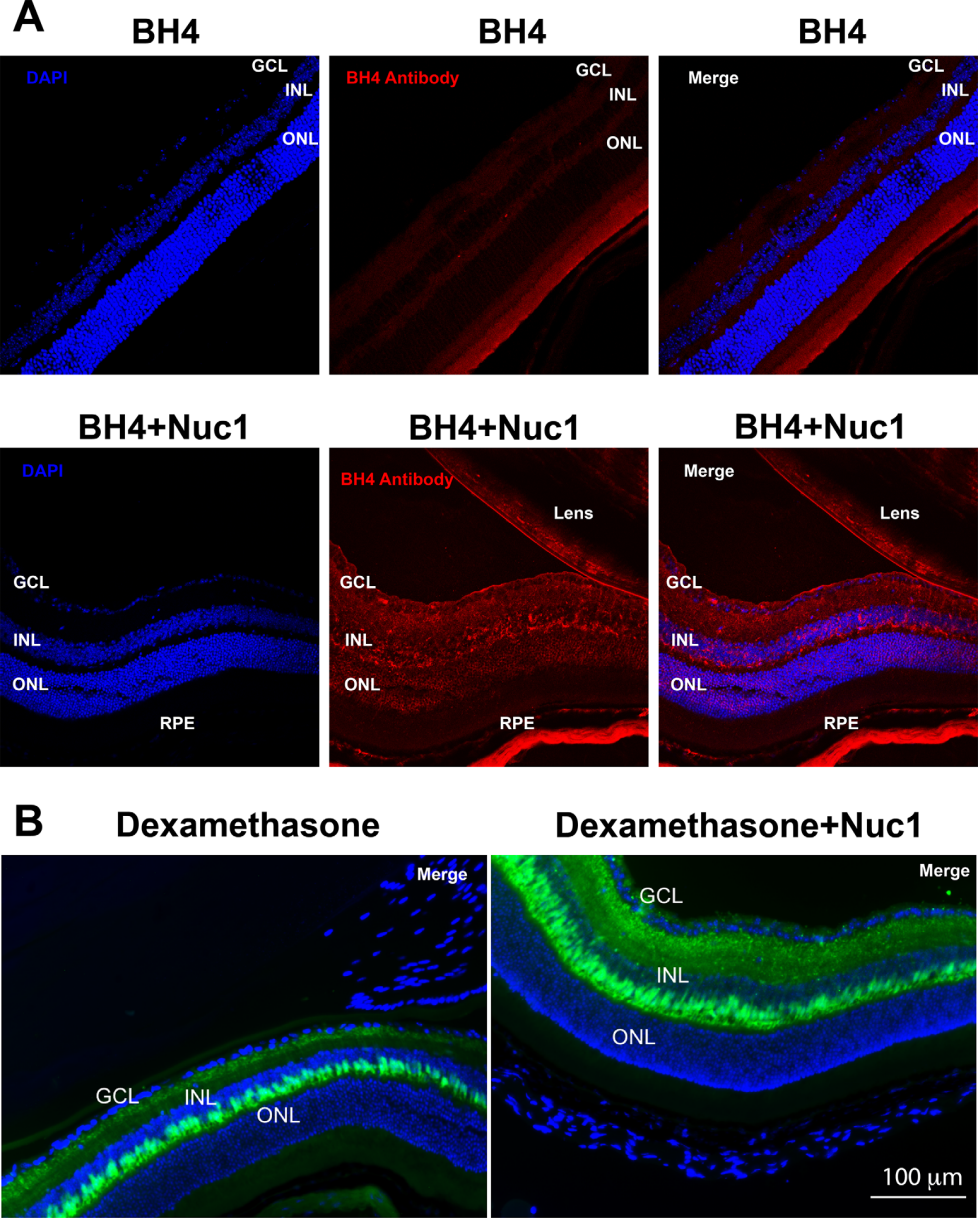
Nuc1 facilitates delivery of peptides and steroids into the retina. (**A**) Representative retinal cryosections from mice at 4 hours postinjection with BH4 alone (*red*) or coinjected with BH4 (*red*) and unlabeled Nuc1 (BH4 + Nuc1) and stained with DAPI (*blue*) and antibody against BH4 shows uptake of BH4 + Nuc1, but not BH4 alone, in the GCL, INL, and ONL. (**B**) Representative retinal cryosections from mice at 4 hours postinjection with fluorescein-labeled dexamethasone alone (*green*) or coinjected with fluorescein-labeled dexamethasone (*green*) and unlabeled Nuc1 (dexamethasone + Nuc1) and stained with DAPI (*blue*) shows enhanced uptake of dexamethasone + Nuc1 relative to dexamethasone alone in inner nuclear and plexiform layers. Representative images are from randomly selected sections obtained from *n* = 3 to 4 animals per group.

Molecules significantly smaller than whole proteins have the potential to act as therapeutic agents. For example, the BH4-domain peptide from Bcl-xL is known to have antiapoptotic activity in vivo.^35^ BH4 is not known to have any significant cell-penetrating properties. Thus, several groups have delivered BH4 to cells by chemical linkage of BH4 linked to the prototypical cell-penetrating peptide HIV TAT.^36^^–^^38^ In order to investigate whether Nuc1 can enhance uptake of the peptide BH4 by the retina, *without* the need for chemical linkage, we injected 6-week-old C57BL/6J mice intravitreally with 4 µg BH4 peptide only or in combination with 4 µg Nuc1. After 4 hours, eyes were harvested and processed as above for cryosectioning. Retinal sections were stained with BH4-recognizing antibody against BCL-xl. As anticipated, we found that the BH4 peptide by itself had limited uptake by the retina. In contrast, when BH4 was combined with Nuc1, there was a marked qualitative increase in uptake of fluorescently labeled BH4 peptide (Fig. 5A), specifically in the GCL, INL, ONL, and RPE.

Small molecules, including steroids, can serve as anti-inflammatory agents, but steroids such as dexamethasone are known to have significant side effects when injected into the vitreous, including the development of cataracts or increased intraocular pressure.^39^^–^^41^ We hypothesized that Nuc1 may facilitate penetration of steroids into tissues, thus potentially enabling a reduction in dosage of steroid needed to reach efficacy. In order to test this hypothesis, we injected either 1 µg fluorescein-labeled dexamethasone alone or 1 µg fluorescently labeled dexamethasone in combination with 1 µg Nuc1 into the vitreous of mice. After 4 hours, eyes were harvested and processed as above for cryosectioning and imaged for fluorescence. We found that both dexamethasone alone and dexamethasone with Nuc1 were taken up by the retina. In both cases, uptake was observed primarily in the inner nuclear and plexiform layers. However, the uptake of dexamethasone was qualitatively greater when it was coinjected with Nuc1 (Fig. 5B).

### Nuc1 Enhances Adeno-Associated Virus Infection of Retinal Cells In Vivo

**Figure 6.**
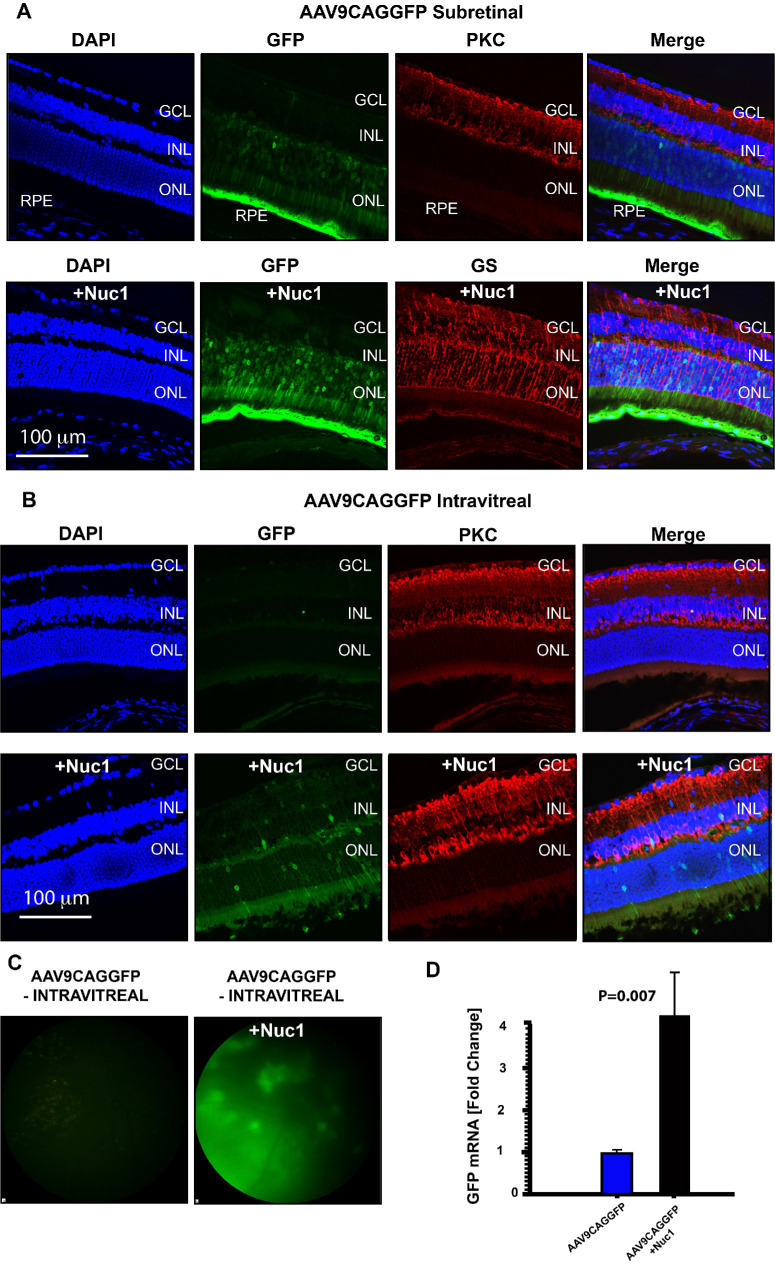
Nuc1 enhances subretinal and intravitreal delivery of AAV2/9 to retinal cells. (**A**) Representative retinal cryosections from mice at 2 weeks postinjection in the subretinal space with AAV2/9-expressing GFP (AAV9CAGGFP) and stained with DAPI (*blue*) and glutamine synthase (*red*; Müller cells) show markedly enhanced transduction by AAV9CAGGFP (+ Nuc1), as determined by GFP expression, in the GCL, INL, and RPE, relative to AAV9CAGGFP alone. In contrast to AAV9CAGGFP alone, which transduces only a few photoreceptors, when AAV9CAGGFP is mixed with Nuc1 prior to injection, GFP expression is also observed throughout the ONL. (**B**) Representative retinal cryosections from mice at 2 weeks after intravitreal injection with AAV9CAGGFP and stained with DAPI (*blue*) and anti-PKC (*red*) show enhanced transduction by AAV9CAGGFP (+ Nuc1), as determined by GFP expression, in the GCL and INL, relative to AAV9CAGGFP alone. In contrast to AAV9CAGGFP alone, when AAV9CAGGFP is mixed with Nuc1 prior to injection, GFP expression is also observed in the ONL. (**C**) Representative fundus images from mice intravitreally injected with AAV9CAGGFP alone or coinjected in the vitreous with AAV9CAGGFP and Nuc1 shows widespread but GFP expression of varying intensity in the retina of coinjected mice. (**D**) Quantitation of GFP mRNA from retinal lysate of intravitreally injected mice shows a 4.3-fold (*P* = 0.007) increase in GFP expression from AAV9CAGGFP when coadministered with Nuc1. Images presented are representative from *n* = 4 to 5 animals. Significance test among the groups was analyzed using an unpaired *t*-test and significance was considered at *P* < 0.05.

As differentiated neurons, retinal photoreceptors are typically difficult to infect with recombinant adeno-associated virus (AAV)—the most commonly used viruses in retinal gene therapy. We hypothesized that Nuc1 may enhance the potency of AAV infection of retinal cells in vivo. To test this hypothesis, we injected 1.1 × 10E9 genome copies of a recombinant AAV serotype 2 (pseudotyped with AAV 9 capsid; AAV2/9) expressing GFP (AAV9CAGGFP) subretinally into the eyes of adult (6- to 8-week-old) C57BL/6J mice with or without 1 µg Nuc1. Two weeks following injection, eyes were harvested and processed for cryosectioning and imaging. We found that although AAV9CAGGFP infected mostly the photoreceptors (ONL) and RPE, when it was coinjected with Nuc1, the expression of GFP was qualitatively superior (Fig. 6A).

When injected intravitreally, expression of GFP from AAV9CAGGFP was not observed in any cells of the inner or outer retina (Fig. 6B). However, we found that combining 1  µg Nuc1 with AAV9CAGGFP prior to injection improved GFP expression via the intravitreal route of administration (Fig. 6B). This pattern of AAV9CAGGFP + Nuc1 transduction was observed across the retina at variable levels of intensity, as documented by fundus photography (Fig. 6C) of live animals. Quantitation of viral infections was performed by RT-PCR, and we found that GFP expression from AAV9CAGGFP was enhanced 4.3-fold (*P* = 0.007) when coadministered with Nuc1 during intravitreal injection (Fig. 6D).

### Nuc1 Penetrates Retinal Cells by Macropinocytosis

**Figure 7.**
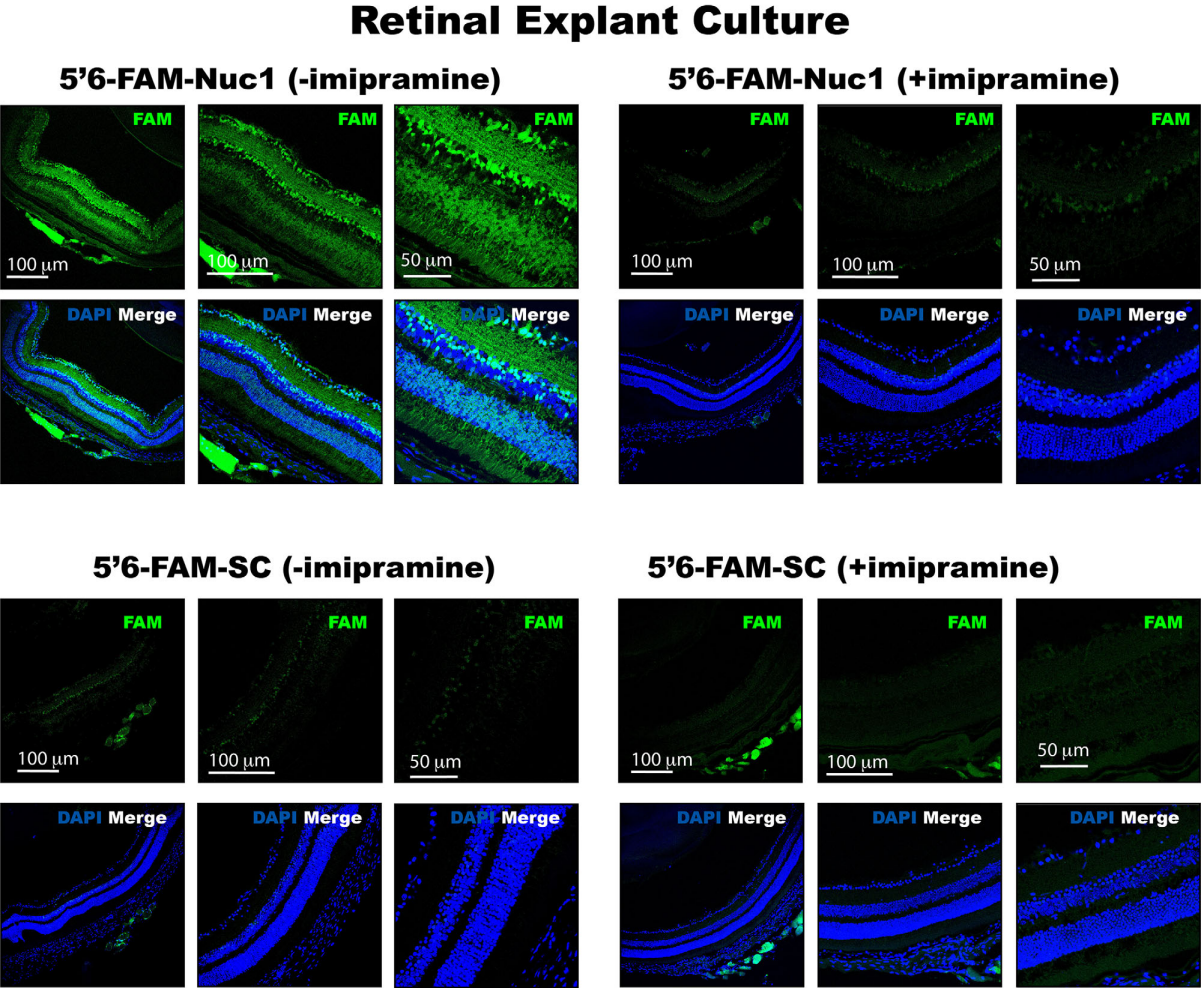
Macropinocytosis plays a role in the uptake of Nuc1. Retinal tissue explants incubated in DMEM/F12 with 5′ 6-FAM-Nuc1 or 5′ 6-FAM-SC (a scrambled peptide), in the presence or absence of 5 µM imipramine, an inhibitor of macropinocytosis, for 4 hours. Explants coincubated in imipramine had significantly reduced uptake of 5′ 6-FAM-Nuc1 and a marginal reduction in uptake of 5′ 6-FAM-SC, suggesting that Nuc1 is internalized into cells using a process involving macropinocytosis. Images presented are representative from the sections obtained from *n* = 3–4 retinal explants per group.

Macropinocytosis is a specialized pathway of endocytosis characterized by the nonspecific internalization of extracellular fluids, solutes, and membrane in large endocytic vesicles known as macropinosomes. Macropinocytosis plays a role in a variety of physiological processes, including nutrient sensing, recycling of plasma proteins, and signaling.^42^^,^^43^ Molecules that bind HSPGs can stimulate bulk cellular uptake via macropinocytosis.^19^ Imipramine is a well-characterized inhibitor of macropinocytosis that does not exert cytotoxic effects in vitro and does not inhibit other endocytic pathways.^44^ In order to test the hypothesis that Nuc1 can be taken up by cells via macropinocytosis, murine retinal tissue explants were incubated in 5′ 6-FAM-Nuc1 (1 µg/mL) in the presence or absence of 5 µM imipramine. After 4 hours, explants were processed for cryosectioning as above. We found robust uptake of 5′ 6-FAM-Nuc1 within 4 hours of incubation (Fig. 7). In contrast, explants coincubated with 5′ 6-FAM-Nuc1 and imipramine had significantly reduced uptake of 5′ 6-FAM-Nuc1 (Fig. 7). The activity of 5′ 6-FAM-Nuc1 was shown to be specific by incubation of retinal explants in 5′ 6-FAM-SC, a scrambled peptide. 5′ 6-FAM-SC did not show any significant uptake relative to Nuc1 or consequentially any significant imipramine-mediated reduction (Fig. 7). We conclude that Nuc1 peptide penetrates retinal cells in part by the process of macropinocytosis.

## Discussion

A variety of CPPs have been previously developed that enable the delivery of biologically active conjugates, including proteins, peptides, DNA, small interfering RNAs (siRNAs), and small-molecule drugs, into cells and tissues.^45^ CPPs have been grouped into a variety of classes: (a) cationic, including HIV TAT, penetratin, or poly arginine; (b) amphipathic, including Transportan and Pep-1; and (c) hydrophobic, including Pep-7.^1^ Although CPPs have been found to have properties useful for delivery of molecules across the negatively charged plasma membrane, the efficiency of individual peptides varies, depending on the tissue being targeted. An important property of Nuc1 relative to other CPPs is its intrinsic ability to efficiently deliver molecules into cells without the need for chemical or electrostatic conjugation with its payload, most likely through a macropinocytic process. This property of Nuc1 significantly differentiates it from prototypical properties of CPPs, and thus Nuc1 may be described as a “chaperone” for macromolecular delivery.

Nuc1 was modeled in part using an approach similar to that of POD—the presence of heparan sulfate on retinal cells and tissues.^3^ The importance of heparan sulfate for CPP function has been described for CPPs known as Vectocell peptides.^46^ Another important property in the modeling of Nuc1 was based on the cell–cell interactions of laminins.^8^ These trimeric proteins can bind to other cell membrane and extracellular matrix molecules. At least seven laminin chains, α3, α4, α5, β2, β3, γ2, and γ3, have been localized to the matrix surrounding photoreceptors and the first synaptic layer where photoreceptors synapse with retinal interneurons.^47^ A region of laminin 1 is an active site for cell adhesion^9^ and known to bind nucleolin,^10^ a protein found on the surface of rapidly dividing cells^11^^–^^13^ but unexpectedly found in the retina, including terminally differentiated cells such as photoreceptors.^14^

While the detailed mechanism(s) of uptake of Nuc1 by retinal cells will be the focus of a future study, development of Nuc1 creates the opportunity to mobilize libraries of previously well-characterized compounds and potential therapeutics without the need for the engineering and characterization of novel chemical conjugates as is the case for CPPs.

Following intravitreal injection, Nuc1 targeted essentially all of the major cell types in the retina, including ganglion cells, bipolar cells, photoreceptors, and Müller cells. In contrast, POD transduction was observed only in ganglion cells and a few cells in the INL following intravitreal injection.^3^ Previously, in our studies with POD, we demonstrated POD–GFP fusion protein delivery only into ganglion cells following intravitreal injection.^48^ Nuc1, on the other hand, is observed to deliver the similar-sized red fluorescent protein**,** mCherry**,** to cells throughout the retina, with the highest rate of transduction of the ONL (photoreceptor nuclei) following intravitreal injection. Again, Nuc1 could deliver mCherry to the ONL without the need for any fusion or chemical linkage between mCherry and Nuc1. Delivery was rapid and easily observable within a 4-hour period. There was no significant mCherry signal in the retina without Nuc1 over this same time. Although staining of the ONL was most prominent, other cell types transduced included ganglion cells, bipolar cells, Müller cells, and cone photoreceptors. The efficient retinal delivery of proteins, without the need for conjugation, suggests the potential application of Nuc1 as a delivery vehicle for not only single but also multiple proteins simultaneously for the treatment of photoreceptor disease.

While the above studies suggested that marker proteins could penetrate the retina, they did not prove that therapeutically relevant proteins could be transported intact across the plasma membrane of cells. Studies demonstrating that functional XIAP could be codelivered with Nuc1 into the photoreceptor cell bodies and inhibit apoptosis was key in demonstrating the functional utility of Nuc1. Previously, we have shown that XIAP may be chemically conjugated to an aptamer that binds nucleolin and such an aptamer–XIAP conjugate can be used to inhibit chemically induced apoptosis in retinal cells.^15^ However, chemical conjugation of XIAP was technically challenging and such protein modifications do not lend themselves to large-scale manufacturing of protein therapeutics.

Apoptosis or programmed cell death is a central pathway in a variety of retinal degenerations, including the heterogeneous disorders termed retinitis pigmentosa, and thus the ability to inhibit apoptosis in the retina has significant potential clinical application.^22^^,^^49^^,^^50^ Retinal degeneration can involve a number of cell death pathways other than apoptosis, including necroptosis and autophagy, as well as inflammation.^51^ Future studies will investigate delivery by Nuc1 of a combination of proteins targeting different pathways involved in photoreceptor cell death.

While the above proteins (mCherry and XIAP) are relatively large**,** at approximately 27 Kd and 55 Kd, respectively, we were surprised to find that penetration of a 150-Kd anti-VEGF antibody could be significantly enhanced when Nuc1 was added to the antibody formulation. This led to an increase in efficacy of the antibody in attenuation of laser-induced CNV. Enhanced drug delivery by Nuc1 may be valuable for improving the potency of drugs, which could facilitate reduction of dosage, toxicity, and cost of goods. Remarkably, and of considerable potential significance, Nuc1 facilitated attenuation of laser-induced CNV by anti-VEGF antibody when applied *topically*. One of the major reasons for patients with age-related macular degeneration continuing to lose vision**,** despite availability of anti-VEGF therapy**,** is lack of compliance with frequent intraocular injections**.** The ability to deliver anti-VEGF antibodies via topical application as eye drops may thus have significant potential utility,^52^ although whether these observations would translate to larger eyes such a nonhuman primates or eventually humans remains to be determined.

Nuc1 also facilitated delivery of small(er) molecules**,** such as peptides. The antiapoptotic peptide BH4 has been utilized in a large number of studies.^35^^–^^38^ However, BH4 does not cross the plasma membrane and is thus generally chemically conjugated to peptides such as HIV TAT. We found that following intravitreal injection, BH4 could penetrate the outer and inner layers of the retina when formulated with Nuc1. The steroid dexamethasone was also found to have markedly increased transduction of inner retinal cells when administered by intravitreal injection with Nuc1. Again, given the toxicity associated with steroids following intravitreal injection, there is significant potential use of Nuc1 to reduce steroid dose.^53^^–^^55^

Finally, we found that Nuc1 enhanced infection of AAV2/9 via both the subretinal and intravitreal routes of delivery. Although the subretinal space is immune sequestered, injection of AAV into the vitreous has been previously found to be immunogenic.^56^^,^^57^ Efforts at modifying AAV have failed to translate toward significant reduction of AAV-associated immune responses following intravitreal injection.^58^ The immune response to intravitreally injected AAV in humans is associated in part with the dose of virus that is administered, and theoretically, improvements in AAV transduction via use of Nuc1 may enable use of a lower dose of virus without compromising efficacy.

While we did not observe any obvious toxicity associated with Nuc1 in these proof-of-concept short-term (4 hours) or longer-term (2 weeks) studies, detailed analysis of toxicity will be the subject of future studies. Furthermore, while sequences used in the design of Nuc1 were selected based on prior studies of nucleolin and heparan-sulfate proteoglycan-binding sequences, detailed analysis of mechanism(s) of uptake is ongoing in our laboratory. Such studies face the challenge of limitations associated with retinal cell culture with a bona fide extracellular matrix.

In summary, in this study, we have described a novel peptide, Nuc1, that can deliver a variety of agents, including small and large molecules into retinal cells. Photoreceptor cells are the main target cell for a large variety of genetic retinal disorders. Highly efficient delivery of functional proteins into photoreceptors to the extent reported here has not been demonstrated previously. The proof-of-concept studies described here justify more detailed studies of Nuc1.
